# Adrenalectomy improves cardiovascular profile in patients with mild autonomous cortisol secretion: extension of a multicenter randomized controlled trial to 12 months

**DOI:** 10.1007/s40618-026-02872-w

**Published:** 2026-04-06

**Authors:** Valentina Morelli, Maria Strata, Serena Palmieri, Carmen Aresta, Vittoria Favero, Sofia Frigerio, Flavia Pugliese, Antonio Musolino, Alberta Dall’Antonia, Sabrina Corbetta, Maura Arosio, Alfredo Scillitani, Iacopo Chiodini

**Affiliations:** 1 Department of Endocrine and Metabolic Diseases,, Istituto Auxologico Italiano, IRCCS, via Ariosto 13, 20145 Milan, Italy; 2 Cardiology Unit, Fondazione IRCCS Ca’ Granda, Ospedale Maggiore Policlinico, Milan, Italy; 3 Unit of Endocrinology, Fondazione IRCCS Ca’ Granda-Ospedale Maggiore Policlinico, Milan, Italy; 4 Unit of Endocrinology, Grande Ospedale Metropolitano Niguarda, Milan, Italy; 5 Department of Medical Biotechnology and Translational Medicine, University of Milan, Milan, Italy; 6 Unità Operativa di Endocrinologia Fondazione IRCCS,, “Casa Sollievo della Sofferenza” – Hospital, San Giovanni Rotondo, Foggia, Italy; 7 Department of Biomedical, Surgical and Dental Sciences, University of Milan, Milan, Italy; 8 Department of Clinical Sciences and Community Health, University of Milan, Milan, Italy

**Keywords:** Adrenal incidentaloma, Mild autonomous cortisol secretion, Cardiovascular risk factors, Adrenalectomy

## Abstract

**Purpose:**

The best therapeutic approach in patients with mild autonomous cortisol secretion (MACS) is debated. In this extension of a randomized controlled trial to 12 months we aimed to evaluate the effect of adrenalectomy on blood pressure, cardiac structure and coagulation factors in outpatients with MACS.

**Methods:**

patients with unilateral adrenal incidentaloma ≥ 1 cm and cortisol after 1 mg dexamethasone suppression test (F-1mgDST) between 1.8 (50 nmol/L) and 5 µg/dL (138 nmol/L) were enrolled and randomized to adrenalectomy (Arm-A) or conservative approach (Arm-B). Blood pressure control, echocardiographic indices and coagulation factors, were assessed at baseline and 12 months after recovery or observation, in Arm-A and Arm-B, respectively.

**Results:**

51 subjects (23/28 in Arm-A/Arm-B) were enrolled. At follow-up the prevalence of blood pressure improvement was higher in Arm-A (10/23 patients, 43.5%) than in Arm-B patients (4/28 patients, 14.3%, p = 0.02). The improvement of blood pressure control was 5.4-fold more frequent in Arm-A patients (CI, 1.16–24.9 p = 0.03), regardless of confounding factors. In Arm-A, left ventricular mass, left atrial area and the prevalence of left atrial dilatation decreased at follow-up (96.4 ± 28.8 vs 87.6 ± 25,6 g/m^2^, p = 0.02; 28.4 ± 9.9 vs 22.6 ± 12.4 cm^2^, p = 0.04, 70.6% vs 35.3% p = 0.04, respectively) whereas all these parameters remained stable in Arm-B. At the end of follow-up, Arm-A patients had a lower prevalence of altered anti-coagulant parameters than Arm-B patients (2/19 patients, 10.5% vs 13/24 patients 54.2%, respectively, p = 0.002).

**Conclusion:**

In patients with MACS, surgery ameliorates blood pressure, cardiac structure, and coagulation factors.

**Trial registration:**

NCT number 04860180.

## Introduction

The adrenal incidentaloma (AI) is an adrenal mass (≥ 1 cm) incidentally discovered during imaging scans performed for unrelated symptoms or suspicions. The prevalence of AI is rising across all age groups, driven by the increased use of advanced imaging technologies. Approximately 80% of AI are benign adenomas. Within this group, 40–70% are non-functioning adenomas, and 20–50% exhibit mild autonomous cortisol secretion (MACS) [1].

MACS was defined in the 2023 guidelines released by the European Society of Endocrinology (ESE) as a mild biochemical cortisol excess without the clinical features specific of hypercortisolism (round face, truncal obesity, easy bruising, thin extremities, proximal myopathy, cutaneous purple striae).

The definition of MACS is based on the evidence of cortisol levels above 50 nmol/L following a 1-mg overnight dexamethasone suppression test (F-1mgDST) [2].

It’s widely recognized that patients with overt endogenous hypercortisolism (Cushing’s syndrome) suffer from excessive morbidity and a 2.5-fold increase in mortality, primarily due to cardiovascular (CV) impairment. Furthermore, mortality risk in patients with Cushing’s syndrome persists even after long-term remission, indicating that hypercortisolism can induce irreversible cardiovascular damage [3]. Recent studies have shown that even patients with a low-grade cortisol excess not only have a higher incidence of different comorbidities but also experience a higher rate of CV events and mortality than patients with non-functioning adenomas [4]. Changes in cardiovascular system produced by cortisol excess have been extensively studied. However, so far, the mechanisms involved in the development of cardiovascular diseases are only partially understood. MACS is further complicated by several comorbidities, including systemic arterial hypertension, visceral obesity, glucose tolerance impairment, type 2 diabetes and dyslipidemia, resulting in a metabolic syndrome that promote left ventricular remodeling and hypertrophy with a consequent diastolic dysfunction [5].

Recent evidence has shown that exposure to MACS causes structural and functional changes of the left ventricle (LV), resulting in LV hypertrophy in up to 24–42% of patients with a higher prevalence of LV diastolic dysfunction (LVDD) when compared to non-functioning adenomas. [6, 7]. Alterations of coagulation state is another trigger point in cardiovascular risk. It is known that patients with Cushing’s syndrome have an increased incidence of thromboembolic complications related to increased concentration of coagulation factors such as factor V, VIII, and Von Willebrand. Similarly, in patients with mild hypercortisolism increased levels of fibrinogen levels and reduced protein C and S have been described together with Von Willebrand factor alterations [8], but data abort the effect of MACS resolution are lacking.

The management of adrenal incidentalomas is currently a matter of debate. Some authors suggested the surgical approach in the presence of at least two complications of cortisol excess [9]. The 2023 ESE guideline remains extremely careful in the management of MACS, suggesting a conservative approach in most cases, especially in patients without relevant cortisol-related comorbidities. In particular, the panel underlines that decision for surgery should be individualized, balancing patient-specific factors with the degree of cortisol autonomy. Surgery may be indicated for MACS patients with a unilateral adrenal mass, and comorbidities that are progressive, difficult to manage, or cause disproportionate end-organ damage. Younger patients (under 65 years), particularly women, are identified as a potential high-risk group who might benefit most from intervention, although this is not yet fully supported by interventional study data [2]. However, to date, an increasing body of high-quality scientific evidence supports the efficacy of surgical approach in improving blood pressure and glycemic control in MACS patients, with the adverse cardiovascular risk profile improving after adrenalectomy [10–13]. The long-term persistence of post-adrenalectomy benefits remains a subject of debate, as recent data indicate a potential waning effect of intervention during follow-up [7]. In patients with bilateral adrenal tumor and MACS unilateral adrenalectomy failed to demonstrate a meaningful effect in reducing cardiometabolic comorbidities [14].

This study reports data from the extension of a previous multicenter randomized trial evaluating the effects of adrenalectomy in a cohort of outpatients with MACS and unilateral adrenal tumor. The presented results focus not only on blood pressure variations but also on the 12-months post–surgical changes in cardiac function and coagulation indices that contribute to cardiovascular risk.

## Methods

### Population, inclusion/exclusion criteria

**Fig. 1:**
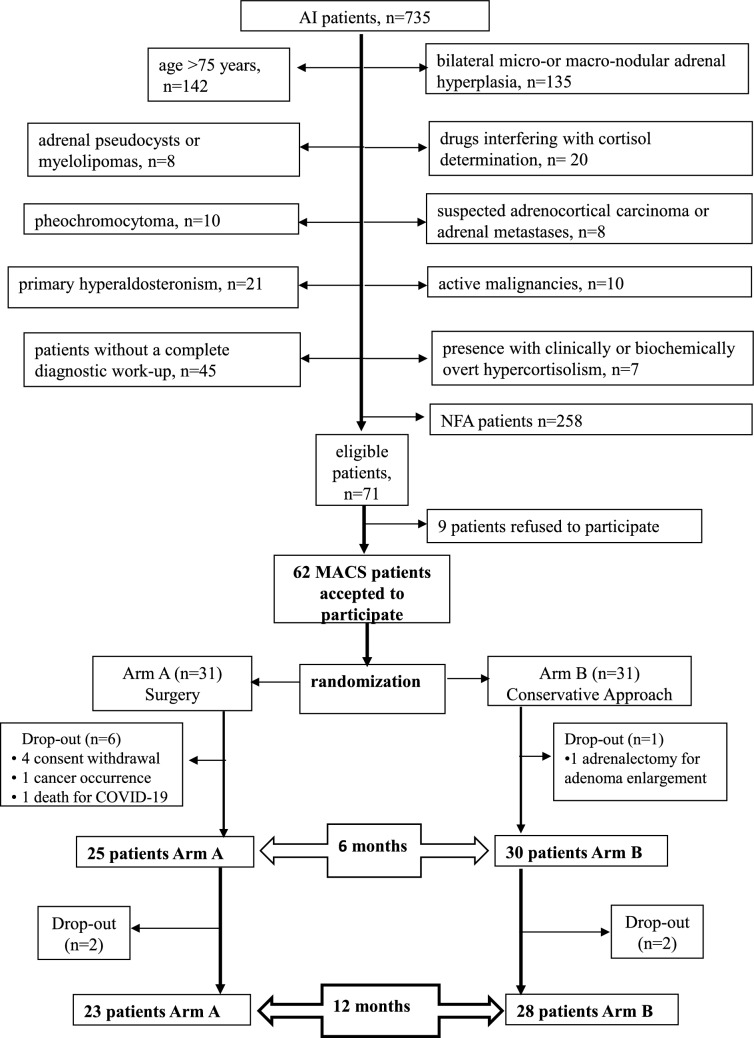
Study enrollment flow-chart. NFA: non-functioning adenomas, MACS: mild autonomous cortisol secretion

The study was approved by the Ethical Committee of Milan Area B (n. 809_2015, resolution n. 516 25/03/2016). In September 2016 the study protocol, identified by the NCT number 04860180, was started to assess the effect of adrenalectomy in patients with MACS. A cohort of 62 consecutive eligible MACS patients (Fig. 1) have been randomized to surgery (Arm-A) or to a conservative approach (Arm-B) to evaluate the effect of adrenalectomy on several outcomes possibly associated with hypercortisolism. The study has been conducted at three different Italian hospitals: Fondazione IRCCS Cà Granda and IRCCS Istituto Ortopedico Galeazzi in Milan and IRCCS “Casa Sollievo della Sofferenza” Hospital in San Giovanni Rotondo, Foggia. The enrolment ended in February 2020. Informed consent was obtained from each patient after explanation of the purpose and nature of all procedures used.

The inclusion criteria were: 1) age between 40 and 75 years; 2) diagnosis by imaging of unilateral AI ≥ 1 cm with radiological features at computed tomography consistent with an adrenocortical adenoma (homogeneous and hypodense, Hounsfield units < 10 or with proven radiological dimensional stability). All patients with confirmed F-1mgDST levels between 1.8 µg/dL (50 nmol/L) and 5.0 µg/dL (138 nmol/L) were eligible for study inclusion. This condition, defined as PACS (possible autonomous cortisol secretion), accordingly to the 2016 ESE Guidelines, at the time of study beginning was considered to be at low cardiovascular risk compared to ACS (autonomous cortisol secretion, defined by cortisol after 1 mg DST > 5.0 µg/dL- 138 nmol/L), and rarely deserving of treatment. However, data were scarce making this category of patients, who can now be defined as having MACS (according to current guidelines) even with cortisol after 1 mg DST < 5.0 µg/d (138 nmol/L), particularly worthy of study [15].

The exclusion criteria were: 1) hypogonadism, thyrotoxicosis, chronic renal failure and hepatic disease, alcoholism, rheumatologic and haematological disease and eating disorders, including binge eating disorder, bulimia nervosa and anorexia nervosa 2) intake of drugs influencing cortisol and dexamethasone metabolism or cortisol secretion; 3) signs or symptoms specific of hypercortisolism; 4) possible metastatic diseases or radiologic appearance not consistent with an adrenocortical adenoma; 5) biochemical evidence of pheochromocytoma and primary aldosteronism; 6) ACTH-dependent hypercortisolism [16]; 7) incomplete diagnostic work-up. Patients with AI larger than 5 cm have been excluded, as in these subjects according to the consensus in force at the time of enrollment, surgery was recommended due to the high risk of malignancy [17].

Data about the 55 patients that completed the 6 months study protocol have been previously published. (11) All Arm A patients underwent laparoscopic adrenalectomy without complications.

In both groups, two patients withdrew the consent after the 6 month’s evaluation due to personal reasons. Eventually, 51 patients (23 in Arm-A and 28 in Arm-B) completed the 12 months extension of the study protocol.

### Study evaluations

At baseline and at 12 months the following variables have been evaluated: adrenal function: i) plasma adrenocorticotrophic hormone (ACTH), 24 h urinary free cortisol (UFC), F-1mgDST levels; ii) physical examination: systolic BP (SBP) and diastolic BP (DBP), body weight (BW) and body mass index (BMI); iii) transthoracic full cardiac ultrasound (Philips IE 33) by a cardiologist blinded to hormonal data, with measurement of LV ejection fraction (EF, %), LV end-diastolic diameter (LVED, mm), interventricular septal thickness (IVS, mm), LV posterior wall thickness (LVPWT, mm), left atrial area (LA area), LV mass index, corrected for body surface area (LVMI, gr/m.^2^); v) echocolor doppler of supra-aortic trunks measuring early-E-wave (E) and late A-wave (A) across the mitral valve, early diastolic tissue velocities of septal and lateral myocardial portions (E’), isovolumetric relaxation time (IVRT) and E/A and E/E’ ratio reflecting the LV filling pressures [18]; iv) coagulation parameters: protein C, protein S, fibrinogen, antithrombin3, prothrombin time (PT), and activated partial thromboplastin time (aPTT) [16].

### Laboratory assays

The plasma ACTH levels at 8:00 am were measured by chemiluminescence (Immulite 2000, Siemens Medical Solutions Diagnostics, Los Angeles, California, normal values 10–55 pg/mL). The serum cortisol concentrations were measured by the second-generation monoclonal immunoassay Elecsys Cortisol II with limit of detection 1.5 nmol/L, limit of quantitation 2.0 nmol/L, interassay CV ranging from 1.9 to 10.1% and intra-assay CV from 1.5 to 5.4% (Elecsys Cortisol immunoassay, Roche Diagnostics, Mannheim, Germany, on CobaS E 602). The UFC determination (reference range up to 43 μg/24 h) was performed by Liquid Chromatography-Tandem Mass Spectrometry (LC–MS/MS, coefficient of variation < 10%, accuracy between 98 and 100%, and limit of quantification 1 µg/L, as previously described [19] and centralized at Fondazione IRCCS Cà Granda, Milan). As per our protocols, the 24-h urine collection has been repeated in case of possible inadequacy (i.e. reduced 24-h urine quantity and/or creatinine excretion). Coagulation parameters were measured with routinely used assays in each centre. The plasma ACTH levels at 8:00 am were measured by chemiluminescence (Immulite 2000, Siemens Medical Solutions Diagnostics, Los Angeles, California, normal values 10–55 pg/mL). The serum cortisol concentrations were measured by the second-generation monoclonal immunoassay Elecsys Cortisol II with limit of detection 1.5 nmol/L, limit of quantitation 2.0 nmol/L, interassay CV ranging from 1.9 to 10.1% and intra-assay CV from 1.5 to 5.4% (Elecsys Cortisol immunoassay, Roche Diagnostics, Mannheim, Germany, on CobaS E 602). The UFC determination (reference range up to 43 μg/24 h) was performed by Liquid Chromatography-Tandem Mass Spectrometry (LC–MS/MS, coefficient of variation < 10%, accuracy between 98 and 100%, and limit of quantification 1 µg/L, as previously described [19] and centralized at Fondazione IRCCS Cà Granda, Milan). As per our protocols, the 24-h urine collection has been repeated in case of possible inadequacy (i.e. reduced 24-h urine quantity and/or creatinine excretion). Coagulation parameters were measured with routinely used assays in each centre.

### Assessment of complications, management and measurement of variations

Blood pressure was measured following the European Society of Cardiology/European Society of Hypertension (ESH/ESC) guidelines as previously described [20]. Arterial hypertension was defined as the detection of SBP) and/or DBP above 140 mm Hg and 90 mm Hg, respectively and/or the need of any antihypertensive treatment [21]. Type 2 Diabetes was diagnosed using the American Diabetes Association current clinical practice recommendations [19–21].

LV hypertrophy was defined if LVMI was > 88 g/m^2^ in women and 102 g/m^2^ in men; LA dilatation was defined in the presence of LS area ≥ 20 cm^2^; relative wall thickness (RWT, normal values > 0.42) was calculated as (IVS + PWT)/LVED [18].

An “Anti-coagulant” pattern was defined in the presence of protein C, protein S or antithrombin 3 above the upper normal limit of the assay used. A “Pro-coagulant” pattern was defied in the presence of protein C, protein S, antithrombin 3 levels below the lower normal limit and fibrinogen levels higher than upper normal limit of the assays used. No patient was taking anti-coagulant treatment. Prevalence of anti-platelet treatment was comparable between the two groups.

At baseline, as previously reported (11), most of the hypertensive patients were under treatment with angiotensin-converting enzyme inhibitors or angiotensin-receptor blockers in monotherapy or in combination with diuretics, calcium antagonists or beta blockers without differences between the two groups. Regarding antidiabetic drugs at baseline, 4 out of 5 diabetic patients of Arm A were under treatment with metformin, 1 patient did not take any drug. Among 5 diabetic patients of Arm B, 3 patients were under treatment with metformin at baseline, 1 patient with DPPIV inhibitor and 1 patient did not take any drug.

After the baseline evaluations, the patients of Arm-A were addressed to surgery. In the Arm-A group, the evaluations started after the post-surgery substitutive corticosteroid therapy withdrawal. In Arm-B, the patients with borderline-elevated blood pressure or grade 1 arterial hypertension, with prediabetes or with overweight were suggested to follow intensive lifestyle behaviour changes, while patients with grade 2 to 3 hypertension, with not fully controlled diabetes (HbA1c > 53 mmol/mol), with obesity or with dyslipidaemia were addressed to cardiologists and/or diabetologists to consider therapy modifications. The medical treatment was not standardized but personalized for obtaining the best possible result in the individual patients [21].

The improvement or the worsening of BP if the non-hypertensive patients passed from a pre-hypertension category to another or the hypertensive patients from an arterial hypertension grade to another [21] or if the dosage and/or number of antihypertensive drugs was at least halved or doubled, respectively. [18, 19].

Variations of cardiac function were defined based on changes of the prevalence of LV hypertrophy or LA dilatation after surgery or observation in Arm-A and Arm-B, respectively.

Variations of coagulation status were defined based on the changes of the prevalence of at least one altered coagulation factor, considered together as “Altered Coagulation” or separately as “Anti-coagulant” or “Pro-coagulant” pattern, after surgery or observation in Arm-A and Arm-B respectively.

### Sample size and statistical analysis

Prior data indicate that the rate of improvement of blood pressure among controls and experimental subjects is 0.13 and 0.68, respectively. Based on these data, the sample size evaluated in the present study consented to have a power of 0.9 (type I error 0.05) [11].

A block randomization was used to reduce bias and achieve balance in the allocation of participants to treatment arms.

Statistical analysis was performed by SPSS version 29.0 statistical package (IBM SPSS, Milan, Italy). Results are expressed as mean ± standard deviations and with range for normally distributed continuous variables or as absolute frequencies and percentages for categorical variables. The comparisons of continuous variables between Arm-A and Arm-B group at baseline and after 12-month follow-up have been performed by t-test. The categorical variables have been compared by using χ2 test or Fisher exact test, as appropriate.

The primary endpoint of the study was to assess the change in BP control (amelioration or worsening) and compare the prevalence of these changes between Arm-A and Arm-B patients.

The secondary endpoints of the study were designed to evaluate changes in echocardiographic indices and measurements of the coagulation state. The paired samples T test was used to compare baseline and 12 months echocardiographic parameters.

The logistic regression analysis was used to assess the independent association between the amelioration or worsening of BP control and the surgical treatment or conservative approach after adjusting for possible confounding variables, such as age, sex, BMI, presence of T2D and arterial hypertension, F-1mgDST levels at baseline. P values < 0.05 were considered statistically significant.

## Results

The comparisons of the characteristics of Arm-A (n = 23) vs Arm-B (n = 28) patients and between baseline and 12-months follow-up are reported in Table 1. At the randomization, Arm-A and Arm-B patients were comparable in terms of age (61.9 ± 11.9 vs 65.9 ± 9.3 yrs, respectively p = 0.17), sex (female/male ratio 16/7 vs 22/6, respectively p = 0.32), diameter of the adenoma (3.2 ± 0.8 vs 2.9 ± 0.7, respectively, p = 0.13), adrenal function parameters, BMI, mean SBP, mean DBP, and prevalence of arterial hypertension, diabetes or IGT/IFG.

**Table 1:** Clinical and biochemical modifications in MACS patients conservatively treated (Arm-A) and surgically treated (Arm-B) and at baseline and at 12 months follow-up

	Baseline			12 months		
	ArmA (n=23)	ArmB (n=28)	p	ArmA (n=23)	ArmB (n=28)	p
BMI (kg/m^2^)	27.6±5.6 (18.6-43.9)	26.7±5.2 (18.7-37.4)	0.59	27.7±5.8 (17.8-39.4)	26.6±5.3 (18.4-39.7)	0.49
ACTH (pmol/L)	1.9±0.6 (1.1-3.0)	2.2±1.1 (1.1-4.4)	0.29	5.7±3.1* (1.3-15.7)	2.7±1.3 (0.3-4.4)	<0.001
F- 1mgDST (nmol/L)	88.3±24.8 (51.3-138)	80.0±22,1 (50-118.7)	0.18	13.8±44.2* (22.1-38.6)	82.8±27.6 (46.9-143.5)	<0.001
UFC (nmol/24 hour)	72.8±34.2 (28.7-140.8)	60.4±27.0 (28.2-118.7)	0.16	59.6±27.3 (13.5-126.1)	76.7±43.6 (18.7-178.8)	0.12
SBP (mmHg)	139±14.1 (100-167)	138.5±13.9 (110-165)	0.82	128.8±14.2 (100-160)	133.7±18.0 (95-160)	0.29
DBP (mmHg)	81.7±10.2 (70-100)	77.1±9.6 (60-97)	0.09	75.7±9.5 (60-95)	75.6±9.6 (50-92)	0.99
Patients with HT, n (%)	16 (69.6)	21 (75.0)	0.67	17 (73.9)	22 (78.6)	0.75
BP control (%) (stable/worse/better)	-	-		10/3/10 (43.5/13.0/43.5)	15/9/4 (53.6/32.1/14.3)	0.02
Patients with DM, n (%)	5 (21.7)	5 (17.9)	0.72	5 (21.7)	7 (25)	0.78
Patients with IGT/IFG, n (%)	7 (38.9)	8 (34.8)	0.79	6 (33.3)	10 (43.5)	0.54
Number of antihypertensive drugs, median (IQR)	2 (2)	2 (2.5)	0.55	1 (2)	2 (2)	0.19

Data are mean ± SD with range in parenthesis or absolute number with percentage in parenthesis. BMI: body mass index; F-1mgDST serum cortisol levels after 1–mg dexamethasone suppression test; UFC: urinary free cortisol (normal values 3-43 μg/24hour); ACTH: adrenocorticotroph hormone (normal values 10-55 pg/mL). IFG: impaired fasting glucose; IGT: impaired glucose tolerance. BP: blood pressure; SBP: systolic BP, DBP: diastolic BP; HT: hypertension; SI conversion factors: F-1mgDST 27.59, ACTH 0.22, UFC 2.759; *P=0.025 vs baseline; IQR, interquartile range

As expected, after surgery ACTH levels significantly increased and F-1mgDST levels decreased in Arm-A patients, and at follow-up these parameters were significantly different from values observed in Arm-B patients, while UFC levels were comparable between the two groups.

**Fig. 2:**
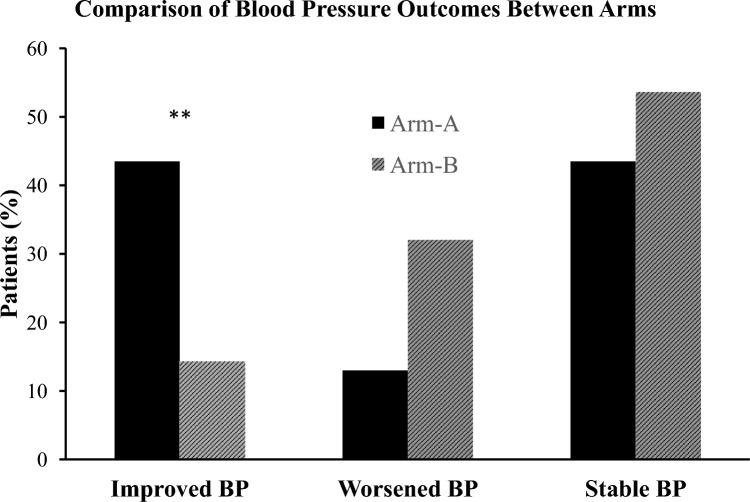
Blood pressure changes at 12 months. BP: blood pressure control, Arm-A: surgically treated group; Arm-B: conservatively treated group; The improvement or worsening during follow-up have been defined as follows: for blood pressure-BP control if the non-hypertensive patients passed from a pre-hypertension category to another or the hypertensive patients from an arterial hypertension grade to another [21] or if the dosage and/or number of antihypertensive drugs was at least halved or doubled, respectively; **:p = 0.020. BP was stable/worse/better in 10/3/10 and 15/9/4 patients respectively in Arm A and Arm B

At follow-up no differences were observed in terms of BMI, and prevalence of arterial hypertension, diabetes or IGT/IFG between the two groups. At 12 months, we observed that the prevalence of BP improvement was higher in Arm-A (10/23 patients, 43.5%) than in Arm-B (4/28 patients, 14.3%, p = 0.02). At variance, worsening of BP control occurred in 13% (3/23) and 32.1% (9/28) of patients in Arm-A and Arm-B patients respectively (p = 0.11) (Fig. 2). In particular in Arm-A in 8/10 hypertensive patients BP levels were reduced, with a good blood pressure control obtained with equal or less antihypertensive therapy. No hypertensive patients had a complete resolution of arterial hypertension. One patient with high-normal blood pressure levels became normotensive. In Arm-B the improvement of BP levels was observed in 3 hypertensive patients and in one patient with borderline BP levels.

The improvement of BP control was 5.4-fold more frequent in operated patients (95% confidence interval-CI, 1.16–24.9, p = 0.03), regardless for age (OR 1.0, 95%CI 0.95–1.1, P = 0.68), sex (OR 2.4 95%CI 0.52–10.8, P = 0.27), F-1mgDST levels (OR 1.3 95%CI 0.47–2.7, P = 0.79) BMI (OR 1.0 95%IC 0.89–1.2, P = 0.64) and presence of arterial hypertension (OR 3.7 95%CI 0.51–28.0, P = 0.19) and diabetes at baseline (OR 1.0 95%CI 0.17–6.2, P = 0.95).

**Fig. 3:**
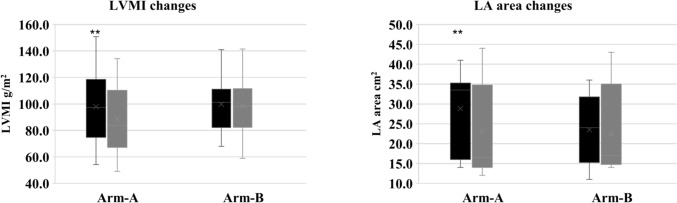
Changes of cardiac structure. Left ventricular mass index (LVMI) and left atrial area (LA area) in surgically treated group (Arm-A, 17 patients) and conservatively treated group (Arm-B, 24 patients) at baseline (black bar) and 12 months follow-up (grey bar). **:p < 0.05

Echocardiographic parameters were obtained in a subgroup of patients (n = 17 in Arm-A and n = 24 in Arm-B) comparable in terms of age (61.7 ± 11.6 vs 66.0 ± 8 yrs respectively, p = 0.16), sex (female/male ratio 10/7 vs 19/5 respectively, p = 0.18), BMI (26.8 ± 4.7 vs 26.5 ± kg/m^2^ respectively, p = 0.85) and F-1mgDST levels (104.7 ± 49.7 vs 88.3 ± 52.4 nmol/L respectively, p = 0.06). At baseline and at 12-months follow-up all the echocardiographic parameters were comparable between Arm-A and Arm-B group (Table 2). However, LVMI and left atrial area (LA-area) decreased at follow-up in patients of Arm-A group whereas both parameters remained stable in Arm-B group (Fig. 3). The prevalence of LV hypertrophy remained stable at follow-up in both groups, whereas the prevalence of LA dilatation decreased from 70.6% (12/17 patients) to 35.3% (6/17 patients) in Arm-A group, p = 0.04.

**Table 2:** Echocardiographic parameters in MACS patients surgically treated (Arm-A) and conservatively treated (Arm-B) and at baseline and at 12 months follow-up

Echocardiographic measurements	Arm-A		p	Arm-B		p
	Baseline(n=17)	12 months (n=17)		Baseline(24)	12 months(24)	
EF (%)	63.6±4.5	62.3±5.1	0.30	64.6±4.7	62.04	0.05
LVED (mm)	46.3±5.8	45.4±5.1	0.30	45.4±3.3	45.3±3.4	0.90
IVS (mm)	9.8±1.8	9.4±1.9	0.14	9.4±2.1	9.4±1.4	0.92
LVPW (mm)	9.6±1.8	9.4±2.0	0.41	9.2±1.6	9.2±1.3	1.00
RWT	0.40±0.07	0.39±0.07	0.41	0.40±0.06	0.40±0.06	1.00
LVMI (gr/mq)	96.4±28.8	87.6±25.6	0.02	93.3±21.5	90.9±19.4	0.50
LV hypertophy, n (%)	10 (58.8)	8 (47.1)	0.50	11 (45.8)	10 (41.7)	0.77
LA area (cm^2^)	28.4±9.9	22.5±12.5	0.04	23.5±9.9	22.6±10.5	0.72
LA dilatation, n (%)	12 (70.6)	6 (35.3)	0.04	11 (45)	8 (33)	0.36
E/E’*	7.9±3.1	8.2±3.1	0.83	9.9±1.9	10.4±2.6	0.58
E/A*	0.9±0.3	0.8±0.2	0.16	0.9±0.3	0.87±0.3	0.19
IVRT (ms)*	92.6±87.9	87.9±20.5	0.57	101.3±19.3	101.5±20	0.97

EF: Left ventricular ejection fraction, LVED: Left ventricle end-diastolic diameter, IVS: interventricular septal thickness, LVPWT: left ventricular posterior wall thickness, RWT: Relative wall thickness, LVMI: Left ventricular mass index, LV hypertrophy: LVMI >88g/m2 in women and 102g/m2 in men. LA area left atrial area. LA dilatation: LS area≥20 cm^2^ (12). E: early wave transmitral diastolic velocity; A late-wave transmitral diastolic velocity; e′, early diastolic velocity of septal and lateral myocardial portions; E/A early/late wave transmitral diastolic velocityratio; E/e′, E-wave transmitral velocity to early diastolic velocity. IVRT: Isovolumetric relaxation time.*These parameters were available in a subgroup of 12 and 18 patients in Arm-A and Arm-B respectively.

Data about coagulation parameters are reported in Table 3. Coagulation parameters were obtained in a subgroup of patients (n = 19 in Arm-A and n = 24 in Arm-B) comparable in terms of age, (64.0 ± 12 vs 66.0 ± 8 yrs respectively, p = 0.43), sex (female/male ratio 13/6 vs 19/5 respectively, p = 0.49), BMI (27.7 ± 6.0 vs 26.5 ± kg/m^2^ respectively, p = 0.43) and F-1mgDST levels (85.4 ± 24.8 vs 88.3 ± 52.4 nmol/L respectively, p = 0.66). At baseline at least one altered parameter of coagulation state was found in the 63% (27/43 patients) of the whole group of MACS patients without differences between Arm-A and Arm-B group. The most frequently altered parameters were AT3 (29.4% of cases: increased in 17.6% and reduced in 11.8% of cases), fibrinogen levels (increased in 19.6% of cases) and Proteins S (15.7% of cases: increased 11.8% and reduced in 3.9% of cases). Compared with Arm-B patients, at follow up Arm-A patients had a lower prevalence of altered coagulation parameters (7/19 patients, 36.8% vs 17/24 patients, 70.8%, respectively, p = 0.03), in particular of the anti-coagulant pattern (2/19 patients, 10.5% vs 13/24 patients, 54.2%, respectively, P = 0.002). Indeed, antithrombin 3 levels and protein S levels normalized respectively in 3 and 2 patients of Arm-A, whereas fibrinogen levels and protein S levels went out of ranges in 2 patients of Arm-B. No correlations were observed between the coagulation parameters and F-1mgDST levels.

**Table 3:** Coagulation parameters in MACS patients surgically treated (Arm-A) and conservatively treated (Arm-B) and at baseline and at 12 months follow-up

	Arm-A (n=19)	Arm-B (n=24)	p	Arm-A (n=19)	Arm-B (n=24)	p
Protein C (PC),	112.1±24.5 (66-170)	108.4±21.4 (61-150)	0.60	115.8±27.2 (68-190)	109.2±18.8 (76-155)	0.36
Protein S (PS),	90.5±20.1 (61-144)	99.0±23.9 (64-177)	0.21	90.8±17.5 (62-128)	103.1±24.2 (64-162)	0.06
Fibrinogen (FBG)	296.2±50.3 (203-394)	321.2±64.8 (231-462)	0.17	302.9±66.1 (216-496)	325.1±66.7 (218-459)	0.28
Anthitrombin3 (AT3),	99.7±14.6 (66-120)	100.2±14.8 (71-120)	0.92	101.3±12.6 (71-123)	103.5±12.6 (81-128)	0.57
Prothrombin time (PT),	0.98±0.09 (0.9-1.2)	1.004±0.07 (0.9-1.1)	0.48	0.97±0.06 (0.9-1.1)	0.99±0.07 (0.9-1.1)	0.14
Activated partial thromboplastin time (aPTT)	0.96±0.06 (0.85-1.06)	0.98±0.05 (0.9-1.06)	0.19	0.097±0.06 (0.95-1.08)	0.97±0.06 (0.86-1.06)	0.95
Altered Coagulation (%)	10/19 (52.6)	15/24 (62.5)	0.52	7/19 (36.8)	17/24 (70.8)	0.03
Pro-coagulant pattern (%)	6/19 (31.6)	10/24 (41.7)	0.50	7/19 36.8	10/24 (41.7)	0.75
Anti-coagulant pattern (%)	5/19 (26.3)	10/24 (41.6)	0.25	2/19 (10.5)	13/24 (54.2)	0.002

Altered Coagulation: at least one altered coagulation factor among PS, PC, FBG, AT3, PT, aPTT. Pro-coagulant pattern: was defined in the presence of reduced PS, PC or AT3 and elevated FBG. Anti-coagulant pattern: was defined in the presence of elevated PS, PC or AT3

**Fig. 4:**
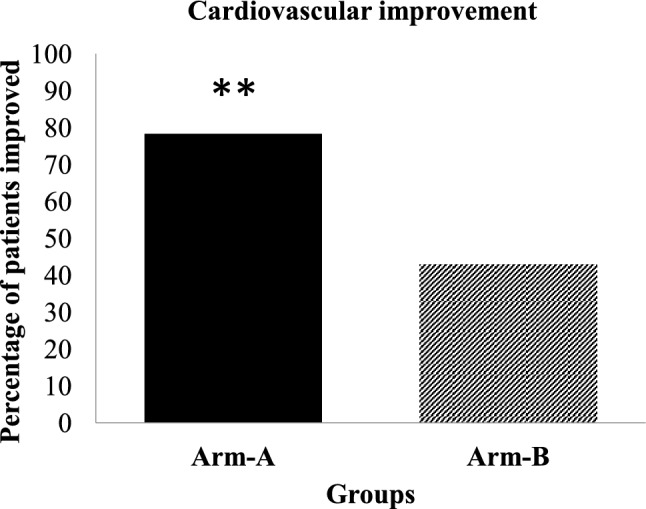
Cardiovascular improvement. Improvement of at least one parameter of cardiovascular risk among arterial hypertension, LV hypertrophy or LA dilatation and altered coagulation in patients of Arm-A compared to Arm-B at follow-up. **: p = 0.011

Considering together arterial hypertension, LV hypertrophy or LA dilatation and coagulation state, the improvement of at least one of these cardiovascular risk factors was observed in 78.3% of patients (18/23) of Arm-A and in 42.9% of Arm-B group (12/28), (p = 0.01) (Fig. 4).

## Discussion

This extension of a randomized multicentre trial shows for the first time that adrenalectomy in patients with MACS leads to a medium-term amelioration not only of BP but also in key cardiovascular parameters, including cardiac function and coagulation markers.

After a 12-months follow-up, patients with MACS who underwent surgical treatment showed a significant BP improvement compared to those managed conservatively. Beyond the reduction in BP, surgical resolution of MACS led to the improvement of LA dilatation, left ventricular indeces and coagulation parameters. In contrast, subjects followed up with a conservative approach tended to experience the worsening of BP more frequently than surgically treated patients. Recent studies have shown that not only patients with Cushing’s syndrome but even patients with a low-grade cortisol excess as MACS suffer from a higher rate of CV events and mortality than patients with non-functioning adenomas [4]. However, the optimal management of AI associated with MACS remains a matter of debate. The ESE guidelines suggests a conservative approach in most cases, unless hormonal excess and/or radiological suspicion of malignancy are present [2]. Other authors advocate for surgery in the presence of at least two complications of cortisol excess, based on evidence suggesting a greater benefit of surgical intervention in reducing CV risk factors [22]. The beneficial effect of surgery on blood pressure and glycemic control has been recently confirmed in three randomized trials [10, 11, 13]. In patients with bilateral adrenal tumor and MACS unilateral adrenalectomy failed to demonstrate a meaningful effect in reducing cardiometabolic comorbidities [14]. In the COAR study the authors reported that among the 118 participants who completed the study with a median follow-up duration of 48 months, the adrenalectomy group exhibited a significantly higher frequency of improved BP (45.7% vs 23.9%) and glucose control (45.7%, vs 15.2%) than the conservative group. Moreover, in the CHIRACIC study Tabarin and co-workers clearly demonstrated that nearly half of the patients who underwent adrenalectomy (46%) no longer needed antihypertensive medications and maintained normal blood pressure at home, with only 15% in the conservative group achieved the same result. Our data are consistent with these findings in terms of the rate of BP improvement (43.5% in Arm-A, 10/23 patients, and 14.3% in Arm-B, 4/28 patients) although in our cohort of patients we did not observe the resolution of arterial hypertension in any of hypertensive patients. This discrepancy could be attributed to the fact that, unlike CHIRACIC study, our research did not include 24-h ambulatory blood pressure monitoring, that would have allowed for a more refined evaluation of BP ameliorations. Notably, in the study by Ueland et. al., at 2-year follow-up the 24-h ambulatory blood pressure monitoring did not demonstrate any blood pressure reduction, in contrast to office blood pressure measurement [13].

Compared with the 6 months follow-up data reported in a previous paper of our group [11], the present results are somewhat different. Indeed, after 6 months of follow-up we found that the surgically treated patients with MACS experienced an amelioration of BP control in 68% of cases (17/25), figures higher than those reported in the present study after a 12 months follow-up. It could be hypothesized that, in some patients the initial improvement of arterial hypertension might be related to a temporary dysfunction of the vaso-regulatory system, which subsequently recovered. Another possible explanation is that a post-surgical hypocortisolism may have played a role, even though the fact that the follow-up observation period started 6 months after that the glucocorticoid substitutive treatment was withdrawn, makes this hypothesis unlikely.

A particularly novel and clinically important finding of our study is the beneficial effect of MACS resolution on cardiac structure and coagulation system, suggesting that the cardiovascular mortality in patients with mild hypercortisolism is not related just to presence of diabetes and arterial hypertension [23–25]. Previous data showed that the exposure to MACS may induce structural and functional changes of the LV, potentially contributing to atrial fibrillation occurrence [6, 7, 26]. Improvement in LVMI and LA dilatation following surgical recovery from MACS suggests a regression of cortisol-induced cardiac remodelling. Moreover, the LVMI reduction without a parallel improvement in LV hypertrophy may indicate that more time is needed for the improvement in quantitative data to translate into improved ventricular function. These structural changes are highly relevant, as both LV and LA dilatation are independent predictors of major CV events beyond the effect of arterial hypertension alone. Thus, surgical benefit likely extends beyond glycemic or BP control [27]. Our findings align with the results reported by De Alcubierre et al. in the ITACA study, which showed an improvement of LV remodeling after adrenalectomy in MACS patients, despite potential differences in some of the outcomes likely attributable to the varying severity of MACS across study populations [7]. Due to the limited 12-months follow-up, we cannot ascertain if the clinical improvement observed in our cohort would eventually taper off at 5 years, mirroring the longitudinal trends reported in the ITACA study.

Additionally, the increased CV risk in MACS patients has been suggested to be related to the alterations of coagulation state, in particular to increased levels of fibrinogen levels and reduced protein C and protein S levels together with Von Willebrand factor alterations [8, 28].

Our data confirm that an alteration of coagulation factors is detectable in more than half of MACS patients. Importantly, we found that 12 months after the recovery from MACS, surgically treated patients had a lower prevalence of altered coagulation parameters in particular of the anti-coagulant pattern as compared with conservatively treated patients. Correction of these abnormalities further contributes to the CV protective effect of adrenalectomy.

Our study has some limitations. Firstly, the limited number of patients that completed the entire follow-up may have concealed some important findings. In particular, the different number of drop-out patients between the two groups may have affected results. Secondly, some data were available only in a subgroup of patients, again potentially limiting the strength of our results. Indeed, due to COVID-19 pandemic restrictions, 6 and 4 patients among Arm-A and Arm-B respectively missed the scheduled examinations. Third, medical treatments among patients of Arm-B were not standardized, potentially introducing a bias. However, all patients with unsatisfactory blood pressure control were evaluated by cardiologists in order to consider the proper therapy modifications according to guidelines. Fourth, the total daily dose of anty-hypertensive medications was not evaluated, therfore, it is not possible to exclude that the two groups at baseline had a different degree of hypertension, affecting study results. Finally, a possible factor that may have limited the significance of the results is the fact that, according to the ESE guidelines in force from 2016 to 2023, only patients with a F-1mgDST between 1.8 (50 nmol/L) and 5 µg/dL (138 nmol/L) (named possible autonomous cortisol secretion) were included, therefore with a more moderate MACS. It can be assumed that if MACS patients with F-1mgDST > 5 µg/dL (138 nmol/L) had also been enrolled, the magnitude of the observed effects could have been greater [15].

Despite these limitations, our data clearly show that at least one CV risk factor (BP, cardiac structure or coagulation) improved in the majority of surgically treated patients.

In conclusion, our findings show that adrenalectomy in MACS patients provides comprehensive CV benefits, improving BP, cardiac structure and coagulation profile. These results support the view that surgery may be an effective strategy for reducing CV risk in selected MACS patients, particularly those with cortisol-related comorbidities, whereas conservative management may be associated with worsening hemodynamic profile.

## Data Availability

Some or all datasets generated during and/or analyzed during the current study are not publicly available but are available from the corresponding author on reasonable request.
